# *In Vivo* Assessment of *NS1-Truncated* Influenza Virus with a Novel SLSYSINWRH Motif as a Self-Adjuvanting Live Attenuated Vaccine

**DOI:** 10.1371/journal.pone.0118934

**Published:** 2015-03-19

**Authors:** John M. Ngunjiri, Ahmed Ali, Prosper Boyaka, Philip I. Marcus, Chang-Won Lee

**Affiliations:** 1 Department of Molecular and Cell Biology, University of Connecticut, Storrs, CT, United States of America; 2 Food Animal Health Research Program, The Ohio State University, Wooster, OH, United States of America; 3 Department of Poultry Diseases, Faculty of Veterinary Medicine, Beni-Suef University, Beni-Suef, 62511, Egypt; 4 Department of Preventive Medicine, The Ohio State University, Columbus, OH, United States of America; 5 Department of Veterinary Bioscience, The Ohio State University, Columbus, OH, United States of America; University of Saskatchewan, CANADA

## Abstract

Mutants of influenza virus that encode C-terminally truncated NS1 proteins (*NS1-truncated* mutants) characteristically induce high interferon responses. The dual activity of interferon in blocking virus replication and enhancing the development of adaptive immune responses makes these mutants promising as self-adjuvanting live-attenuated influenza vaccine (LAIV) candidates. Yet, among the *NS1-truncated* mutants, the length of NS1 is not directly correlated with the interferon-inducing efficiency, the level of attenuation, or effectiveness as LAIV. Using quantitative *in vitro* biologically active particle subpopulation analysis as a tool to identify potential LAIV candidates from a pool of *NS1-truncated* mutants, we previously predicted that a *NS1-truncated* mutant pc2, which was less effective as a LAIV in chickens, would be sufficiently effective as a LAIV in mammalian hosts. In this study, we confirmed that pc2 protected mice and pigs against heterologous virus challenge in terms of preventing clinical signs and reducing virus shedding. pc2 expresses a unique SLSYSINWRH motif at the C-terminus of its truncated NS1. Deletion of the SLSYSINWRH motif led to ~821-fold reduction in the peak yield of type I interferon induced in murine cells. Furthermore, replacement of the SLSYSINWRH motif with the wildtype MVKMDQAIMD sequence did not restore the interferon-inducing efficiency. The diminished interferon induction capacity in the absence of the SLSYSINWRH motif was similar to that observed in other mutants which are less effective LAIV candidates. Remarkably, pc2 induced 16-fold or more interferon in human lung and monkey kidney cells compared to the temperature-sensitive, cold-adapted Ann Arbor virus that is currently used as a master backbone for LAIVs such as FluMist. Although the mechanism by which the SLSYSINWRH motif regulates the vaccine properties of pc2 has not been elucidated, this motif has potential use in engineering self-adjuvanting *NS1-truncated*-based LAIVs.

## Introduction

Influenza virus causes a highly contagious disease in both humans and animals. Morbidity and mortality due to influenza virus infections in humans are accompanied by significant economic losses in terms of healthcare costs and lost labor time, including time absent from work and reduced productivity while at work [1]. Equally important is the impact of this virus on the agricultural economy. Outbreaks of avian influenza often lead to the depopulation of literally millions of chickens, and cost millions of dollars for decontamination and replacement of flocks [2–5]. Swine influenza virus infection causes high morbidity, weight loss and poor growth leading to considerable economic losses around the world [6, 7]. Furthermore, avian and swine influenza viruses are known to contribute to outbreaks and pandemics in humans [5, 8, 9]. Thus, there is an urgent need to develop effective measures to control influenza viruses of diverse origin in human and animals.

Control of influenza is primarily through vaccination with inactivated vaccines to induce antibodies which mainly block the binding of the globular head of HA to the sialic acid receptors on cell surface. A major shortcoming of these vaccines is that the antibody-binding sites of HA naturally undergo mutation at a high rate thereby producing variants which are antigenically distinct from the vaccine strain. Consequently, inactivated vaccines are generally protective against the homologous strains contained therein but not antigenic variants that frequently emerge during the influenza season. This could explain why a recent metastudy of vaccination trials between 1967 and 2011 found that the current influenza vaccines are on average 67% effective in humans [10]. Therefore, new vaccines with increased effectiveness against the target strains as well as cross-protection against heterologous (miss-matched) strains need to be developed. In this context, live attenuated influenza vaccine (LAIV) is known to elicit a broad long lasting immunity by stimulating mucosal (T-cell and secretory IgA) and systemic (IgG) responses that are cross protective against heterologous viral infection [11–14]. However, the broadened immune response afforded by the conventional LAIV over the inactivated vaccine was only seen in the younger cohorts [15, 16] demonstrating the need to improve LAIV without compromising their safety.

One promising approach is to use mutant viruses that encode C-terminally truncated NS1 proteins (*NS1-truncated* mutants) and are attenuated in avian and mammalian hosts. NS1 is an antagonist of interferon induction [17]. In this regard, *NS1-truncated* mutants induce higher type I interferon responses relative to those induced by the parental wildtype strains [17–26]. The dual activity of interferon in blocking replication of influenza virus [21, 27] and enhancing the development of adaptive immune responses [28–37] makes *NS1-truncated* mutants promising as self-adjuvanting live-attenuated influenza vaccine (LAIV) candidates. Yet, among these mutants, the relationship between the size of NS1 and interferon-inducing efficiency or effectiveness as LAIV is complex [18, 19, 21–26, 38]. Through quantitative *in vitro* analysis of biologically active particle subpopulations, we observed that *NS1-truncated* mutants that induce high peak yields of interferon and are efficient as LAIVs also generate large subpopulations of defective-interfering (DI) particles that enhance the efficiency of IFN-inducing particles through a mechanism that presumably blocks the synthesis of viral polymerase protein [21, 22].

Using the *in vitro* biologically active particle subpopulation analysis as a tool to identify potential LAIV candidates from a pool of *NS1-truncated* mutants, we previously predicted that a mutant termed pc2, which is less effective as a LAIV in chickens [38], would be sufficiently effective in mammalian hosts [22]. Herein, we tested the validity of that prediction and found that pc2 protected mice and pigs against heterologous challenge viruses. Also, we show that the SLSYSINWRH motif at the C-terminus of the truncated NS1 protein is important for generation of a large DI particle subpopulation which correlates with enhanced IFN responses and effectiveness of pc2 as a LAIV candidate in mammalian hosts.

## Materials and Methods

### Animals and ethics statement

All animals were maintained, vaccinated, challenged and euthanized in accordance with protocols #2009A0100R and #2008A0210R approved by The Ohio State University Institutional Animal Care and Use Committee (IACUC). These protocols comply with the U.S Animal Welfare Act, Guide for Care and Use of Laboratory Animals and Public Health Service Policy on Humane Care and Use of Laboratory Animals. The Ohio State University is accredited by the Association for the Assessment and Accreditation of Laboratory Animal Care International (AAALAC).

### Immunization and challenge studies in mice and pigs

For the mouse experiment, 6–8 week-old C57BL/6 mice (n = 10 per group) obtained from Jackson Laboratory (Bar Harbor, ME) were mock-vaccinated or inoculated intranasally with 10^6.0^ EID_50_/mouse (diluted in PBS to a final volume of 50 μL) of pc2 (H7N3) or pc4 (H7N3). The latter mutant was previously predicted to be less effective as a LAIV candidate in mammalian hosts [22]. All three groups of mice were challenged intranasally with a heterologous virus strain A/CK/NJ/150383–7/02 (H7N2) (10^6.0^ EID_50_/mouse) three weeks after vaccination. The A/CK/NJ/150383–7/02 virus shares 87.5% amino acid sequence similarity in the HA1 protein with the pc2 or pc4 strain and shows at least 4-fold difference in serologic reactivity in hemagglutination inhibition (HI) test. Mice were monitored daily for clinical signs and weight loss until 14 days post-infection. Blood samples were collected before challenge and also at the end of the experiment. HI test was conducted as previously described [39]. Five mice per group were euthanized at 3 days post-challenge and nasal wash and lung samples were collected. Virus titer was quantified by real time reverse transcription PCR (RRT-PCR) [40] and also titrated in MDCK culture [39].

For the swine experiment, Cesarean-section delivered colostrum-deprived conventional Large White Duroc crossbred pigs were used to minimize passive acquisition of influenza virus-specific maternally derived antibodies. Two reassortant viruses with *NS* gene segments from the original A/TK/OR/71-based pc2 and pc4 viruses [38], respectively, in the backbone of the swine-origin A/TK/OH/313053/04 (H3N2) virus [41] were generated using reverse genetics as described below. Four-week-old pigs (n = 4 per group) that were seronegative to influenza virus were mock-vaccinated with PBS or vaccinated intranasally with 10^6^ EID_50_/ml/nares of pc2 (H3N2) or pc4 (H3N2). At 3 days and 3 weeks post vaccination, sera were collected to measure IFN-α and HI antibody titers, respectively. Pigs were challenged intratracheally with 10^6.5^ TCID_50_ (diluted in PBS to a final volume of 1 ml) of heterologous virus (H3N2 variant, A/SW/OH/FAH9–1/12). The A/SW/OH/FAH9–1/12 virus shares about 93.8% amino acid sequence similarity (based on partial sequence) in the HA1 protein with the pc2 or pc4 strain and shows at least 8-fold difference in serologic reactivity in HI test. Nasal swabs were collected at 3 and 6 days post challenge and two pigs per group euthanized for evaluation of gross lung lesions and to measure virus replication in the lung.

### Humane endpoints in animal experiments

Mice were humanely euthanized when the percentage of bodyweight loss reached 30%. Pigs were humanely euthanized when they had non-responsive anorexia for more than 48 hours after samplings (swabbing, bleeding, anesthesia, and Intra-tracheal inoculation) or when determined moribund by veterinary examination.

### Cells, media and viruses

Simian Marc-145 and murine L(Y) cells were cultivated as described previously [22]. A549 and MDCK cells were grown in DMEM + 10% Fetal Bovine Serum. The following viruses were described previously: A/CK/NJ/150383–7/02 (H7N2), A/TK/OR/71 (H7N3), *NS1-truncated* pc2 and pc4 mutants [38], and A/TK/OH/313053/04 (H3N2) virus [41]. Both pc2 and pc4 were previously confirmed to be genetically stable through plaque purification followed by serial passages in 10-day-old embryonated chicken eggs [38]. The A/Ann Arbor/6/60 (H2N2) virus was kindly provided by Dr. Kanta Subbarao, NIAID, NIH [Comm. with Dr. Philip I Marcus, deceased September 1, 2013]. The swine influenza virus, A/SW/OH/FAH9–1/12, was obtained from depository maintained at Food Animal Health Research Program (FAHRP), The Ohio State University. To make working stocks, the A/CK/NJ/150383–7/02, A/TK/OR/71, pc2, pc4, A/TK/OH/313053/04 and A/Ann Arbor/6/60 viruses were grown in 10-day-old SPF embryonated chicken eggs. The A/SW/OH/FAH9–1/12 was propagated in MDCK cells. All virus stocks were frozen at −80°C until use.

### Induction and bioassay of type I IFN

Induction and bioassay of type I IFN in Marc-145 and L(Y) cells was done as previously described [21, 22]. For optimal production of IFN, about 10^6^ Marc-145 or L(Y) cells were seeded in 50-mm (diameter) dish and incubated at 37.5°C for 9- or 4- days, respectively. Subsequently, a series of cell monolayers were infected with increasing doses of virus suspended in serum-free medium to a final volume of 300 μL. Following attachment at 37.5%°C for 1 h, the virus inoculum was removed and 2 mL of serum-free medium added back. The plates were incubated for a further 20–22 h after which the supernatant medium was harvested and acid labile proteins precipitated with 0.15M perchrolic acid at 4°C for 24 h. The supernatant containing the acid stable type I IFN was clarified by centrifugation and neutralized to pH ~7. Biologically active IFN was detected and quantified using a vesicular stomatitis virus-induced cytopathic effect protection bioassay on confluent monolayers in 96-well plates.

### Detection and quantification of DI and type I IFN-inducing particles

The DI and type I IFN-inducing particles were measured based on their ability to cause gain or loss of specific biological responses as previously described [20, 42]. As a general rule, virus particles encounter host cell receptors through random collisions. When a population of susceptible cells is exposed to a virus suspension, by chance, some cells are not infected, and others receive one particle, two particles, three particles, etc. The number of virus particles that adsorb to and enter any given cell in the population is described statistically by the Poisson distribution.

The biological assay and quantification of DI particles was conducted as detailed in our previous report [42]. Briefly, MDCK cell monolayers were simultaneously exposed to a fixed multiplicity of DI particle-free virus and increasing amounts of the stock preparation being tested for DI particle activity. Virus attachment was as described above. Fresh serum-free DMEM was added and the plates incubated at 37.5°C for 24 h. To prevent super-infection and preserve the initial input multiplicity for accurate measurement of DI particle activity, virus replication was restricted to a single cycle by excluding trypsin in the medium during this incubation period. The viral supernatants were harvested, clarified of cell debris by centrifugation and activated with trypsin (1.5μg/ml) for 30 min at 37°C before virus quantification by plaque assay in chicken embryo kidney cells [42]. To determine the DI particle multiplicity *a posteriori*, the surviving fraction of virus yield (*f*) was plotted as a function of virus dose and best fitted with the following Poisson distribution model: *f* = *e* ^-*m*^, where *m* is the multiplicity of DI particles and *e* is the base of natural logarithm. Then, DI particle titers were calculated as a product of *m*, the factor of virus dilution, and number of cells exposed to the virus [42]. A similar approach was used to quantify the IFN-inducing particles except that the Poisson distribution best-fit models accounted for the fact that an increase in virus dose led to gain in IFN yields until a peak was reached when every cell in the monolayer was infected with at least one inducing particle. These models are detailed in our previous reports [21, 22].

### PCR mutagenesis of the SLSYSINWRH motif

**Fig 1:**
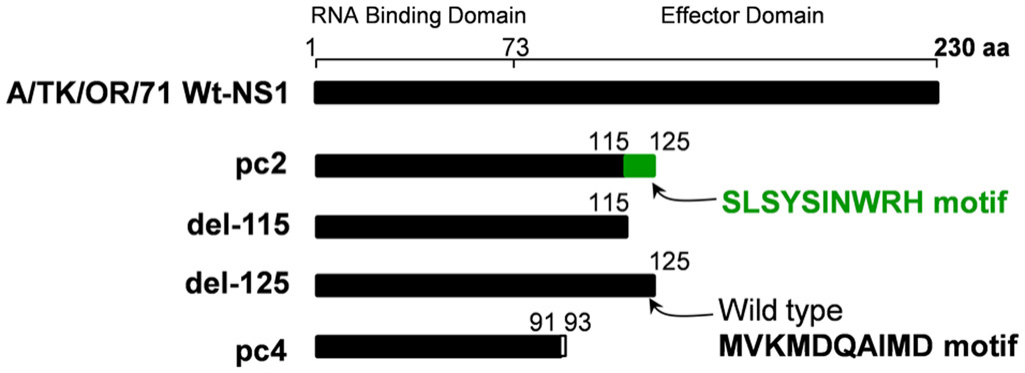
Schematic diagram of NS1 proteins encoded by wildtype A/TK/OR/71 virus, its variants pc4 and pc2 that were tested as candidate live-attenuated vaccines in chickens [38] and mammalian hosts (see text herein), and two pc2 mutants del-115 without the c-terminal SLSYSINWRH motif and del-125 with the SLSYSINWRH motif replaced with the MVKMDQAIMD motif of the wildtype virus. The latter two were generated through PCR mutagenesis of the pc2 virus.

The truncated NS1 protein expressed by pc2 contains a C-terminal SLSYSINWRH motif that is not consensus with the corresponding sequences in the wildtype virus (Fig. 1). We used a pHH21 plasmid with the wildtype *NS* segment as the template to engineer premature stop codons in the NS1 gene in order to generate a mutant without the C-terminal SLSYSINWRH motif (del-115) or with the SLSYSINWRH motif replaced with the MVKMDQAIMD motif of the wildtype virus (del-125). For each mutant, two sets of primers were used to PCR-amplify 5′ and 3′ fragments of the *NS* segment with an overlapping stop codon in the NS1 gene and BsmBI restriction sites. The fragments were then cut with BsmBI restriction enzyme (New England Biolabs Inc., Ipswich, MA), ligated first to make a complete *NS* segment, and finally re-cloned into the pHH21 plasmid vector between RNA polymerase I promoter and terminator sequences as previously described [40]. The following primers were used for PCR mutagenesis: del-115, 5′ fragment—Forward 5′- TAT TCG TCT CAG GGA GCA AAA GCA GG-3′, Reverse 5′- ATA TCG TCT CGT CAC TCA CAG ACC CCC TGT-3′; del-115, 3′ fragment—Forward 5′- ATA TCG TCT CGT ATT AGT AGA AAC AAG G-3′, Reverse 5′- TAT TCG TCT CGG TGA AAA TGG AT-3′; del-125, 5′ fragment—Forward 5′- TAT TCG TCT CAG GGA GCA AAA GCA GG-3′, Reverse 5′- ATA TCG TCT CGT ATT AGT AGA AAC AAG G-3′; del-125, 3′ fragment—Forward 5′- ATA TCG TCT CGT CCT TCA GTC CAT AAT GGC-3′, Reverse 5′ TAT TCG TCT CGA AGA GGA TAA CA 3′.

### *De novo* generation of *NS1-truncated* mutants through reverse genetics

Both del-115 and del-125 viruses were generated *de novo* using the genetic backbone of A/TK/OR/71 (H7N2) virus as previously described [40]. The *NS1-truncated* mutants used in the swine study were similarly generated by inserting the original A/TK/OR/71-based pc2 and pc4 *NS* gene segments [38] in the backbone of the swine-origin A/TK/OH/313053/04 (H3N2) virus [41]. Briefly, 293T cells were co-transfected with the pHH21 plasmid carrying the mutated *NS* segment and the remaining plasmids required for transcription of the full set 8 viral RNA segments. The transfection mixture also contained plasmids for transcription of NP, PA, PB1, and PB2 mRNAs to supply the proteins required for influenza virus replication. Following incubation at 37°C for 48h, supernatants were harvested and inoculated in 10-day-old embryonated chicken eggs to prepare high titer stocks for the *in vitro* biological characterization described herein. The virus was sequenced to confirm the mutated *NS1* genes.

### Statistical analysis

Results are expressed as the mean ± standard deviation where applicable. One-way ANOVA with Dunnett’s Post Test was performed using GraphPad Prism version 5.00 for Windows (GraphPad Software, San Diego California USA) to compare virus replication in mice. Comparisons were considered significantly different at a probability of *p*<0.05.

## Results

Based on a high type I IFN-inducing efficiency that accompanied a biologically active particle composition with a high ratio of DI to IFN-inducing particles when tested in mammalian cell cultures, we previously predicted that pc2 would be more effective than other *NS1-truncaion mutants* as a LAIV candidate in mammalian hosts [22]. To test this hypothesis, *in vivo* studies were conducted in mice and pigs.

### Protective efficacy in mice

All vaccinated mice seroconverted by 3 weeks post vaccination with mean ± SD Log_2_ HI antibody titers of 6.7 ± 0.9 and 7.5 ± 0.7 for pc2- and pc4-vaccinated mice, respectively, which were not significantly different (P>0.05). None of mice in the control group seroconverted. The safety of these live vaccines was monitored by daily observation of the animals and no clinical signs or behavioral changes were observed compared to mock-vaccinated control mice. Further, as expected based on our previous chicken study [38], the average body weight of pc2- or pc4-vaccinated mice was indistinguishable with that of control mice demonstrating that these vaccines are highly attenuated *in vivo*.

**Fig 2:**
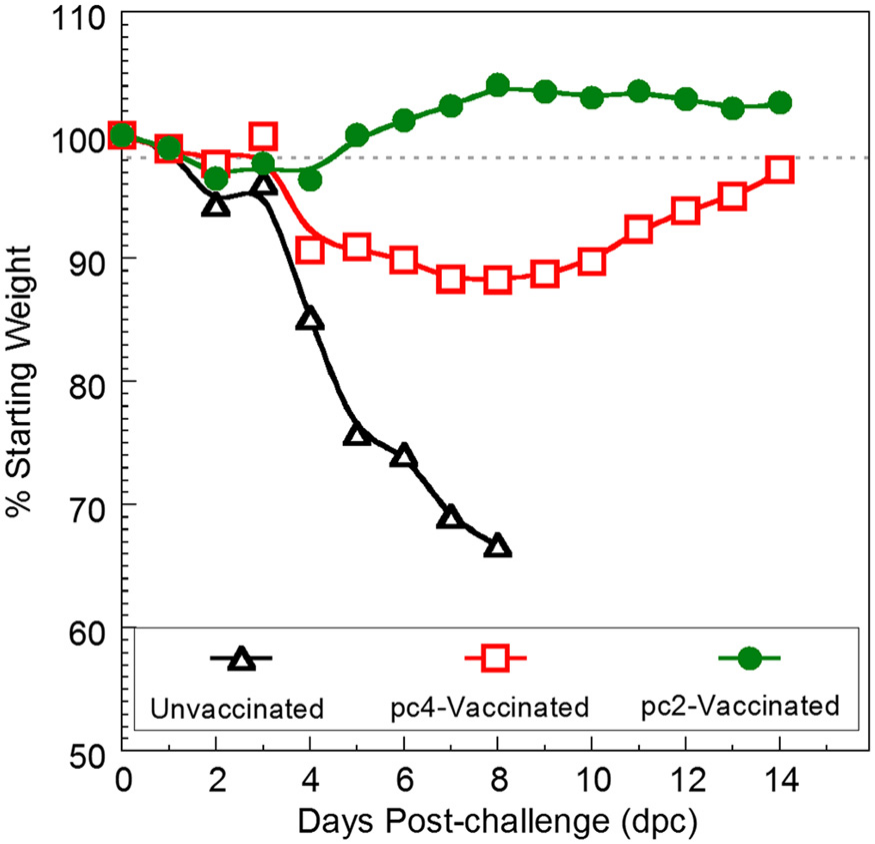
Bodyweight changes after challenge at 2 week post-vaccination. The percent weight loss is based on the average weight at 0 day post-challenge (dpc). Unvaccinated mice were euthanized 8 dpc due to severe weight loss.

After challenge with the heterologous virus, unvaccinated mice rapidly lost their weight and were consequently euthanized at 8 days post-challenge (dpc) (Fig. 2). Mice vaccinated with pc4 lost about 12% of initial body weight by 8 dpc and then recovered most of it by 14 dpc indicating that pc4 was partially protective against the heterologous challenge virus. In a marked contrast, pc2-vaccinated mice gained and maintained weight at 3–4% above the starting weight demonstrating that pc2 was highly protective against weight loss.

Replication and shedding of the challenge virus was quantified by real time reverse transcription PCR (RRT-PCR) and tissue culture at 3 dpc. As expected, none of the unvaccinated mice was protected against the challenge virus as high viral titers were detected in the lung homogenate and nasal wash samples. In contrast, the challenge virus was detected in only 2 to 3 of the 5 mice that received either pc2 or pc4 indicating that these vaccines were partially protective at 3 dpc. Yet, pc2 was significantly more effective at reducing replication and shedding of the challenge virus compared to pc4 (Table 1) reflecting the difference seen in the ability of these vaccines to protect against weight loss (Fig. 2). Altogether, the weight loss and challenge virus replication and shedding data demonstrate that, in mice, pc2 is more effective than pc4 as a LAIV candidate.

**Table 1:** Replication and shedding of A/CK/NJ/150383–7/02 (H7N2) heterologous challenge virus in mice.

Samples	Virus Titers* at 3 days post challenge					
	Control		pc2-vaccinated		pc4-vaccinated	
	RRT-PCR	Tissue culture	RRT-PCR	Tissue culture	RRT-PCR	Tissue culture
**Nasal Wash**	4.4±0.1 (**5/5**)^†^	3.1±0.2 (**5/5**)	1.1±0.3^a, c^ **(**3/5**)	0.0±0.0^b, b^ (**0/5**)	2.8±1.6^c, c^ (**3/5**)	2.4±0.2^a, b^ (**2/5**)
**Lung Homogenate**	3.2±1.1 (**5/5**)	4.0±0.4 (**5/5**)	0.8±0.1^b, b^ (**2/5**)	2.0±0.3^b, b^ (**2/5**)	3.5±1.6^c, b^ (**3/**5)	2.9±0.1^a, a^ (**3/5**)

* Average viral titers are expressed as TCID_50_/ml equivalent by RRT-PCR or TCID_50_/ml by tissue culture ± standard deviation.

^†^Number of positive mice / total number of mice tested.

** Significance differences are indicated for each group as compared to control group and to the other treatment group and P values are as follows:

^a^ Significant difference (P<0.01);

^b^ Significant difference (P<0.001);

^c^ Not-significant difference (P>0.05).

### Protective efficacy in pigs

**Fig 3:**
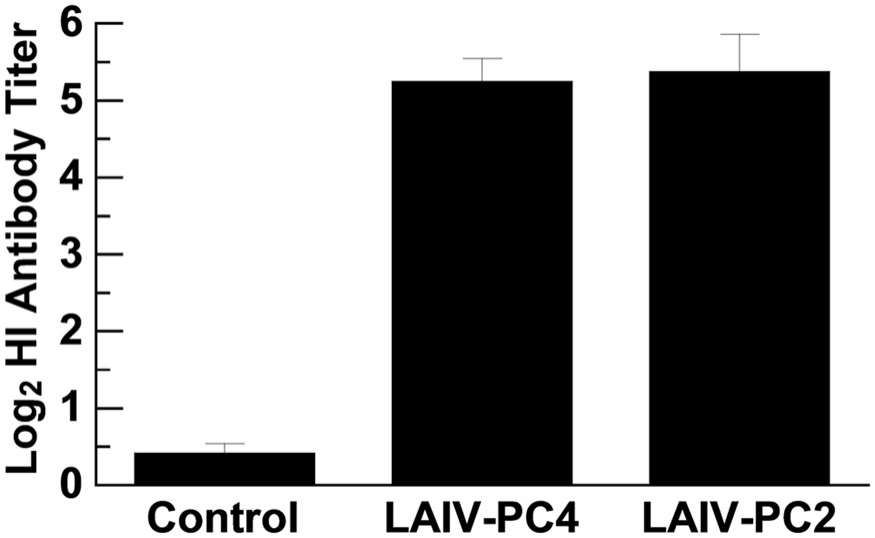
Antibody (Ab) responses induced in pigs. HI Ab titers at 3 weeks post vaccination. Error bars represent the standard deviation from the mean (n = 4).

To further validate the reliability of our *in vitro* screening approach, we carried out a swine study using reassortant viruses that contain *NS* gene segments from the original A/TK/OR/71-based pc2 and pc4 viruses [38], respectively, in the backbone of the swine-origin A/TK/OH/313053/04 (H3N2) virus. As observed in the mice experiment, all vaccinated pigs had influenza virus-specific antibodies (Abs) at 3 weeks post vaccination. The HI Ab titers against the heterologous challenge virus were similar for both vaccine candidates (Fig. 3).

In this experiment, both pc2 and pc4 vaccinated pigs did not show clinical signs or develop pulmonary lesions while unvaccinated-challenged pigs had nasal discharge and showed gross purple-red consolidated pulmonary lesions typical of swine influenza virus infection (S1 Fig.). Although we were unable to differentiate the level of protective efficacy of the two vaccine candidates based on clinical signs or lung pathology following challenge with a heterologous virus, in terms of reducing shedding or replication of the challenge virus in the lungs and nasal cavity, pc2 was more protective than pc4 as indicated by the lesser amount of virus detected from fewer pigs at 3 and 6 dpc (Table 2).

**Table 2:** Replication and shedding of A/SW/OH/FAH9–1/12 (H3N2) heterologous challenge virus in pigs.

Samples	Control		pc2-vaccinated		pc4-vaccinated	
	3 dpc	6 dpc	3 dpc	6 dpc	3 dpc	6 dpc
Nasal Wash	3.62±0.3* (**4/4**)^†^	3.34±0.8 (**2/2**)	2.61±0.0 (**1/4**)	1.76±0.0 (**1/2**)	2.26±1.1 (**2/4**)	3.15±0.0 (**1/2**)
Lung Homogenate	4.94±0.1 (**2/2**)	4.90±1.1 (**2/2**)	2.10±0.0 (**1/2**)	0.0 (**0/2**)	3.20±0.9 (**2/2**)	0.0 (**0/2**)

* Average viral titers were quantified by RRT-PCR and expressed as TCID_50_/ml equivalent ± standard deviation.

^†^Number of positive pigs / total number of pigs tested. dpc: days post-challenge.

### SLSYSINWRH motif alters growth characteristics and plaque phenotype of pc2

Both pc2 and pc4 LAIV candidates were selected by passaging a *NS1-truncated* mutant of A/TK/OR/71 virus in embryonated chicken eggs followed by plaque purification in tissue culture and *de novo* reconstruction through reverse genetics [38, 40]. Each of these viruses has a natural internal deletion in the *NS1* gene that leads to a frame shift and introduction of a premature stop codon. The deletion in pc2 also introduced a 10 amino acid SLSYSINWRH motif at the C-terminus of the truncated NS1 that is not consensus with the corresponding wild type residues at positions 116–125 (Fig. 1). Is this motif important for the biological characteristics observed *in vitro* and therefore a determinant of pc2 effectiveness as LAIV? We mutated the *NS1* gene and, through reverse genetics, successfully rescued two viruses: a mutant without the C-terminal SLSYSINWRH motif, del-115; and a mutant with the SLSYSINWRH motif replaced with the MVKMDQAIMD motif of the wildtype virus, del-125. The NS1 of variant pc4 is shown for comparison (Fig. 1).

**Fig 4:**
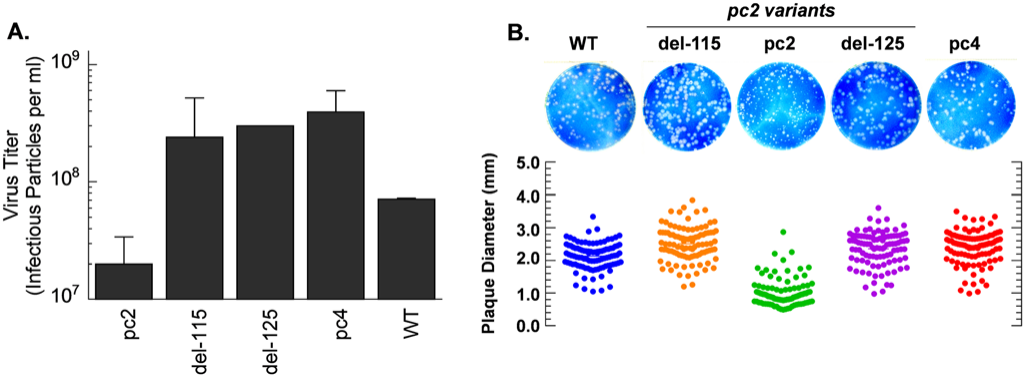
(A) Virus replication in embryonated chicken eggs. Following incubation of infected eggs at 34°C for 72 hours, the chorioallantoic fluid was harvested and infectious virus quantified by plaque assay. Virus titers represent an average of two independent experiments. Error bars represent standard deviation. (B) Plaque morphology in monolayers of chicken embryo kidney cells following incubation at 37°C for 48 hours and Giemsa staining. The lower panel shows the distribution of plaque diameters.

To assess the effect of the SLSYSINWRH motif on replication, the pc2 variants were grown in 9-day old embryonated chicken eggs. Each virus was inoculated in 5 eggs at a dose of 100 infectious particles per egg. Fig. 4A shows that pc2 grew to the lowest titer at 34°C for 72 hours compared to the other viruses. However, all viruses formed plaques of heterogeneous sizes in primary chicken kidney cell monolayers with mean (± SD) plaque diameter of 1.0 (0.4), 2.5 (0.5), 2.4 (0.5), 2.4 (0.5) and 2.1 (0.4) for pc2, del-115, del-125, pc4 and the WT virus, respectively (Fig. 4B). The average diameter of pc2 plaques was significantly smaller compared to the other viruses (P < 0.05). Notably, the plaque size profiles of del-115 and del-125 mutants were similar to those of pc4 and WT. Together, data presented in Fig. 4 demonstrate that the SLSYSINWRH motif was responsible for reduced replication and hence the small plaque phenotype of the pc2 virus.

### SLSYSINWRH motif has potential to regulate LAIV effectiveness

**Fig 5:**
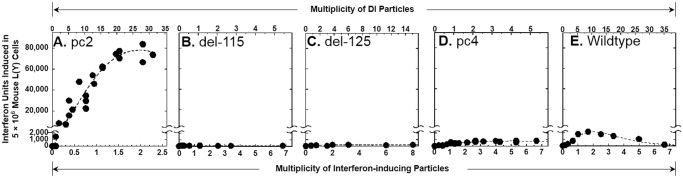
Comparison of IFN induction in L(Y) cells as a function of multiplicity of IFN-inducing particles and defective-interfering particles. The multiplicity of IFN-inducing particles was determined by fitting Poisson distribution curves as described previously [20]. Defective-interfering particles were quantified through virus yield-reduction assays as described previously [42].

Effective *NS1-*based LAIV candidates are characterized by induction of high peak yields of IFN *in vitro* [21, 22]. Thus, we assessed the potential effect of the SLSYSINWRH motif on LAIV effectiveness by comparing IFN induction in murine L(Y) cells. Full IFN induction dose-response curves were generated as described previously [21, 22] to enable determination of the peak yields of IFN as well as the multiplicity of IFN-inducing particles delivered by each virus dose. The original pc2 induced a peak yield of 78,000 U of IFN (Fig. 5A). This yield decreased 821-fold to 95 U when the SLSYSINWRH motif was deleted (Fig. 5B, see del-115). Replacement of the deleted SLSYSINWRH motif with the MVKMDQAIMD motif of the wildtype virus did not restore the efficiency of interferon induction since the peak yield induced by del-125 was 200 U (Fig. 5C). The peak yields of IFN induced by the engineered variants of pc2 (del-115 and del-125) were in the range of those induced by the less effective LAIV candidate pc4 (800 U) (Fig. 5D) and the wildtype virus (2130 U) (Fig. 5E) [22]. Additional cell lines were tested to determine whether the IFN response enhancing activity of the SLSYSINWRH motif was restricted to the murine host. In simian and human host cells, pc2 was still the best IFN inducer but its inducing ability was markedly reduced by deletion or replacement of the SLSYSINWRH motif (Table 3). Thus, the SLSYSINWRH motif was responsible for the enhanced IFN responses induced by pc2. Interestingly, the peak yields induced by pc2 in these cell lines were more than 16-fold higher relative to those induced by the temperature-sensitive cold adapted A/Ann Arbor/6/60 (H2N2) virus that is currently used as a master backbone for LAIVs such as FluMist (Table 3).

**Table 3:** Comparison of peak yields of IFN induced in different mammalian cell lines.

Candidate LAIV	Peak Yield of IFN (Units induced in 5 × 10^6^ Cells)		
	Mouse L(Y)	Human Lung A549	Monkey Marc-145
pc2	78,000	5000	6380
Del-115	95	2480	1115
Del-125	200	2500	1215
pc4	800	495	370
*ts ca* A/Ann Arbor	Not tested	300	260

The dramatic role of SLSYSINWRH motif in enhancing IFN induction prompted us to test whether this motif also affects the balance between DI and IFN-inducing particle subpopulations which we previously showed to regulate IFN induction by *NS1-truncated* mutants [21, 22]. Fig. 5A shows that the original pc2 had a DI to IFN-inducing particle subpopulation ratio of 16—compare input multiplicities of the DI particles with the corresponding input multiplicities of IFN-inducing particles (upper and lower abscissae, respectively). Remarkably, there was a noticeable drop in the subpopulation ratio (3 or lower) when the SLSYSINWRH motif was deleted or replaced with the MVKMDQAIMD motif of the wildtype virus (compare Fig. 5A, B, and C). Therefore, the SLSYSINWRH motif promoted generation of virus composition with a high DI to IFN-inducing particle subpopulation ratio which in turn promoted induction of a high IFN response (Fig. 5) as described previously [22]. Taken together, these data suggest that the SLSYSINWRH motif is essential for the enhanced protection afforded by pc2 when used as a candidate LAIV in mammalian hosts.

## Discussion

Due to loss of NS1 function, *NS1-truncated* mutants of influenza virus induce higher amounts of IFN compared to the related wildtype strains [17]. The immediate role of type I IFN is to signal up-regulation of hundreds of antiviral genes [43] that collectively attenuate the virus by blocking its replication. Another important role of type I IFN is to orchestrate the early stages of the adaptive immune response by signaling the host immune effector cells necessary for the induction of mucosal secretory-IgA, systemic IgG and T-cell responses [28 – 37]. Thus, not only does the enhanced capacity to induce IFN make *NS1-truncated* mutants attenuated in IFN competent hosts [44], it also renders these mutants promising as self-adjuvanting LAIV candidates.

A handful of studies have compared the biology of engineered mutants that lack the entire NS1 gene (delNS1) or encode truncated fragments of NS1 protein containing the N-terminal 73, 99, or 126 (73-del, 99-del, and 126-del, respectively) amino acid residues. These studies demonstrated that the relationship between the size of truncated NS1 protein, IFN-inducing efficiency and attenuation is complex. In this regard, delNS1 mutants (that lack the entire NS1 gene) induce the highest amount of type I IFN and are consequently the most attenuated. However, the behavior of partial deletion mutants (73-del, 99-del, and 126-del) is contrary to this observation—that is, the efficiency of IFN induction and consequently virus attenuation increase with increase in the size of NS1 [18 – 26]. It should be noted that for *NS1-truncated* mutants to be effective immunogens they must self-adjuvant by inducing sufficient amounts of IFN and at the same time not be over-attenuated. Despite having high IFN-inducing efficiency delNS1 mutants are over-attenuated and not suitable for use as LAIVs [45]. In an effort to develop *NS1-*based LAIV candidates with an optimal balance between immunogenicity and safety, we selected several mutants through serial low inoculum passages of TK/OR/71-NS1 [1–124 aa] (H7N3) in embryonated eggs followed by plaque purification and *de novo* reconstruction through reverse genetics [38]. Unlike the delNS1 virus, none of these natural mutants is over-attenuated. However, like their genetically engineered counterparts, the natural mutants displayed a complex relationship between the size of NS1 and immunogenicity [38].

A major goal of our work is to establish novel *in vitro* screening protocols to select the best vaccine candidates and therefore reduce the number of animals required in LAIV efficacy studies. To this end, we carried out comprehensive *in vitro* analysis of biologically active particle subpopulations and found strong evidence that *NS1-truncated* mutants with a subpopulation composition containing a high DI to IFN-inducing particle ratio not only induced high peak yields of IFN [21, 22], they were also more effective as LAIV candidates [38]. On this basis, we predicted that variant pc2, which has a high defective-interfering to IFN-inducing particle ratio and induces a high peak yield of IFN in mouse L(Y) cells (Fig. 5) [22], would be more effective than pc4 when tested as a LAIV candidate in mice. Clearly, pc2 demonstrated superior efficacy to pc4 in terms of protecting mice against weight loss (Fig. 2) and replication of the heterologous challenge A/CK/NJ/150383–7/02 (H7N2) virus (Table 1). A small swine study was subsequently conducted to further examine the *in vivo* characteristics of these vaccines in mammalian species. Serum titers of IFN-α were 100-fold more in pc2-vaccinated pigs compared to the pc4-vaccinated group suggesting that the IFN-inducing phenotypes observed in mammalian cell lines [22] were preserved *in vivo* (S2 Fig.). Although the two vaccines were hardly distinguishable in pigs in terms of protection against clinical signs or lung pathology following heterologous virus challenge (S1 Fig.), pc2-vaccinated pigs shed less virus compared to the other two experimental groups (Table 2). Further work should focus on optimizing LAIV efficacy in pigs and elucidating the level of protective efficacy afforded by pc2. Of note, although the pc2 and pc4 *NS* genes were identical for both mice and swine studies, they were in the respective genetic backgrounds of avian (A/TK/OR/71) and swine (A/TK/OH/313053) viruses. Determination of how compatibility between pc2-*NS* and the background viral genes may affect the vaccine phenotype is a subject of future study. Taken together, the mice and swine experiments presented herein and our previous study in chickens [38] demonstrated that the most efficacious host-tailored vaccine can be selected among *NS1-*based LAIV candidates using *in vitro* analysis as previously suggested [21, 22].

The *NS1-truncated* pc2 is comparable to genetically engineered mutants that lack the entire NS1 gene (delNS1 mutants) in terms of high IFN-inducing efficiency in mammalian host cells [44]. However, unlike pc2 which is moderately attenuated in IFN competent host systems including embryonated chicken eggs (Fig. 4), delNS1 mutants are not only difficult to grow in eggs and cell culture but also unsuitable for use as LAIVs because they are over-attenuated [44, 45]. With this in mind, we conducted *in vitro* mutagenesis to determine whether the non-consensus SLSYSINWRH motif at the C-terminus of the truncated NS1 protein (Fig. 1) contributes to the unique ability of pc2 to induce high levels of IFN without getting over-attenuated. Our data demonstrate that not only did this motif control pc2 replication (Fig. 4), it was also required for generation of a subpopulation composition comprising a high DI to IFN-inducing particle ratio which we previously found to enhance IFN induction by *NS1-truncated* mutants (Fig. 5 and Table 3) [22]. Interestingly, pc2 induced several folds more IFN in different mammalian cells compared with the temperature-sensitive cold adapted A/Ann Arbor/6/60 (H2N2) virus (Table 3) that is currently used as a master backbone for LAIVs such as FluMist. Taken together, these data strongly suggest that modulation of NS1 function by the SLSYSINWRH motif results in a LAIV candidate with an optimal balance between attenuation and immunogenicity. We speculate that a pc2-derived LAIV would be superior to FluMist in terms of immunogenicity and efficacy which should be further validated by *in vivo* study.

We have not yet elucidated the mechanism by which the SLSYSINWRH motif controls the vaccine properties of pc2. Nevertheless, it is known that the functional form of NS1 is homodimeric due to interactions between domains in both N and C terminal regions of the protein [46, 47, 48]. Further, C-terminal truncation of NS1 can cause loss of dimerization ability [26]. It is possible that the SLSYSINWRH motif causes some structural changes that destabilize dimerization of the truncated NS1 further. Another possible mechanism of influencing the above-mentioned biological functions is by autoregulation of the truncated NS1 expression. Studies using engineered *NS1-truncated* mutants that compared expression of multiple viral proteins demonstrated that the C-terminally truncated versions of NS1 are expressed less efficiently relative to full-size (wildtype) NS1 versions [23, 24, 49]. Even among *NS1-truncated* mutants, expression of longer NS1 proteins has been associated with high IFN inducing efficiency and attenuation [23, 24]. Since pc2 had the highest level of NS1 expression among four variants previously tested as LAIV candidates [38], we think it is less likely that the SLSYSINWRH motif-mediated modulation of pc2 vaccine properties is through control of NS1 expression. Regardless of the mechanism of action, more work is planned to determine whether attachment of the SLSYSINWRH motif at the end of truncated NS1 proteins can confer the pc2-like vaccine properties to other *NS1-truncated* mutants.

In this study, we demonstrated for the first time that a motif of non-consensus amino acids (SLSYSINWRH) at the C-terminus end of the truncated NS1 encoded by pc2 virus is critical in determining the vaccine phenotype *in vitro*. Taken together, our unique *in vitro* analysis tool provides a platform for a rapid cost-effective screening of effective LAIVs for specific host and the fine-tuning needed to improve the effectiveness of live vaccines.

## Supporting Information

S1 Fig Gross lung lesions in unvaccinated (left) compared to healthy lungs in pc2 (right) and pc4 (middle) vaccinated pigs at 6 days post challenge with heterologous virus. Areas of purple-red consolidation indicative of pneumonia are shown with arrows in the unvaccinated pig lungs.
(DOCX)

S2 Fig Serum IFN-α titers at 3 days post vaccination. Error bars represent the standard deviation from the mean (n = 4).
(DOCX)

## References

[1] Molinari NA, Ortega-Sanchez IR, Messonnier ML, Thompson WW, Wortley PM, Weintraub E, et al The annual impact of seasonal influenza in the US: measuring disease burden and costs. Vaccine. 2007;25:5086–5096. 17544181 10.1016/j.vaccine.2007.03.046

[2] Bean WJ, Kawaoka Y, Wood JM, Pearson JE, Webster RG. Characterization of virulent and avirulent A/chicken/Pennsylvania/83 influenza A viruses: potential role of defective interfering RNAs in nature. J Virol. 1985;54: 151–160. 3973976 10.1128/jvi.54.1.151-160.1985 PMC254772

[3] Capua I, Mutinelli F, Pozza MD, Donatelli I, Puzelli S, Cancellotti FM. The 1999–2000 avian influenza (H7N1) epidemic in Italy: veterinary and human health implications. Acta Trop. 2002;83:7–11. 12062787 10.1016/s0001-706x(02)00057-8

[4] Cyranoski D. Outbreak of chicken flu rattles Hong Kong. Nature. 2001;412:261 11460124 10.1038/35085715

[5] Fouchier RAM, Schneeberger PM, Rozendaal FW, Broekman JM, Kemink SAG, Munster C, et al Avian influenza A virus (H7N7) associated with human conjunctivitis and a fatal case of acute respiratory distress syndrome. Proc Natl Acad Sci USA. 2004;101:1356–1361. 14745020 10.1073/pnas.0308352100 PMC337057

[6] Van Reeth K, Ma W. Swine Influenza Virus Vaccines: To Change or Not to Change- That’s the Question. Curr Top Microbiol Immunol. 2012;370:173–200. 10.1007/82_2012_266 22976350

[7] Vincent AL, Ma W, Lager KM, Janke BH, Richt JA. Swine influenza viruses a North American perspective. Adv Virus Res. 2008;72:127–54. 10.1016/S0065-3527(08)00403-X 19081490

[8] Beigel JH, Farrar J, Han AM, Hayden FG, Hyer R, et al Avian influenza A (H5N1) infection in humans. N Engl J Med. 2005;353: 1374–1385. 16192482 10.1056/NEJMra052211

[9] Neumann G, Kawaoka Y. The first influenza pandemic of the new millennium. Influenza Other Respi Viruses. 2011;5(3):157–66. 10.1111/j.1750-2659.2011.00231.x 21477134 PMC3073629

[10] Osterholm MT, Kelley NS, Sommer A, Belongia EA. Efficacy and effectiveness of influenza vaccines: a systematic review and meta-analysis. Lancet Infect. Dis. 2012;12:36–44. 10.1016/S1473-3099(11)70295-X 22032844

[11] Gorse GJ, Campbell MJ, Otto EE, Powers DC, Chambers GW, Newman FK. Increased anti-influenza A virus cytotoxic T cell activity following vaccination of the chronically ill elderly with live attenuated or inactivated influenza virus vaccine. J. Infect. Dis. 1995;172:1–10. 7797897 10.1093/infdis/172.1.1

[12] LaMere MW, Lam HT, Moquin A, Haynes L, Lund FE, Randall TD, et al Contributions of antinucleoprotein IgG to heterosubtypic immunity against influenza virus. J. Immunol. 2011;186:4331–4339. 10.4049/jimmunol.1003057 21357542 PMC3159153

[13] Nelson KM, Schram BR, McGregor MW, Sheoran AS, Olsen CW, Lunn DP. Local and systemic isotype-specific antibody responses to equine influenza virus infection versus conventional vaccination. Vaccine. 1998;16:1306–1313. 9682395 10.1016/s0264-410x(98)00009-7

[14] Powell TJ, Strutt T, Reome J, Hollenbaugh JA, Roberts AD, Woodland DL, et al Priming with cold-adapted influenza A does not prevent infection but elicits long-lived protection against supralethal challenge with heterosubtypic virus. J. Immunol. 2007;178:1030–1038. 17202366 10.4049/jimmunol.178.2.1030

[15] Edwards KM, Dupont WD, Westrich MK, Plummer WD Jr, Palmer PS, Wright PF. 1 A randomized controlled trial of cold-adapted and inactivated vaccines for the prevention of influenza A disease. J Infect Dis. 1994;169: 68–76. 8277200 10.1093/infdis/169.1.68

[16] Ohmit SE, Victor JC, Rotthoff JR, Teich ER, Truscon RK, Baum LL, et al Prevention of antigenically drifted influenza by inactivated and live attenuated vaccines. N Engl J Med, 2006;355: 2513–2522. 17167134 10.1056/NEJMoa061850 PMC2614682

[17] Richt JA, García-Sastre A. Attenuated influenza virus vaccines with modified NS1 proteins. Curr. Top. Microbiol. Immunol. 2009;333:177–195. 10.1007/978-3-540-92165-3_9 19768406

[18] Kochs G, Koerner I, Thiel L, Kothlow S, Kaspers B, Ruggli N, et al Properties of H7N7 influenza A virus strain SC35M lacking interferon antagonist NS1 in mice and chickens. J Gen Virol. 2007;88:1403–1409. 17412966 10.1099/vir.0.82764-0

[19] Martínez-Sobrido L, Lienenklaus S, Weiss S, García-Sastre A, Staeheli P. Strong interferon-inducing capacity of a highly virulent variant of influenza A virus strain PR8 with deletions in the NS1 gene. J. Gen. Virol. 2009;90:2990–2994. 10.1099/vir.0.015727-0 19726611 PMC2887554

[20] Marcus PI, Rojek JM, Sekellick MJ. Interferon induction and/or production and its suppression in influenza viruses. J Virol. 2005;79:2880–2890. 15709007 10.1128/JVI.79.5.2880-2890.2005 PMC548469

[21] Marcus PI, Ngunjiri JM, Sekellick MJ, Wang L, Lee CW. In Vitro Analysis of Virus Particle Subpopulations in Candidate Live-Attenuated Influenza Vaccines Distinguishes Effective from Ineffective Vaccines. J Virol. 2010;84: 10974–10981. 10.1128/JVI.00502-10 20739541 PMC2953188

[22] Ngunjiri JM, Lee CW, Ali A, Marcus PI. Influenza virus interferon-inducing particle efficiency is reversed in avian and mammalian cells, and enhanced in cells co-infected with defective-interfering particles. J. Interferon Cytokine Res. 2012;32:280–285. 10.1089/jir.2011.0102 22385205

[23] Quinlivan M, Zamarin D, García-Sastre A, Cullinane A, Chambers T, Palese P. Attenuation of equine influenza viruses through truncations of the NS1 protein. J Virol 2005;79:8431–8439 15956587 10.1128/JVI.79.13.8431-8439.2005 PMC1143746

[24] Solórzano A, Webby RJ, Lager KM, Janke BH, García-Sastre A, Richt JA. Mutations in the NS1 protein of swine influenza virus impair anti-interferon activity and confer attenuation in pigs. J Virol 2005;79:7535–7543 15919908 10.1128/JVI.79.12.7535-7543.2005 PMC1143661

[25] Steel J, Lowen AC, Pena L, Angel M, Solórzano A, Albrecht R, et al Live attenuated influenza viruses containing NS1 truncations as vaccine candidates against H5N1 highly pathogenic avian influenza. J Virol. 2009;83:1742–1753. 10.1128/JVI.01920-08 19073731 PMC2643794

[26] Wang X, Basler CF, Williams BRG, Silverman RH, Palese P, García-Sastre A. Functional replacement of the carboxy-terminal two thirds of the influenza A virus NS1 protein with short heterologous dimerization domains. J Virol 2002;76:12951–12962 12438621 10.1128/JVI.76.24.12951-12962.2002 PMC136679

[27] Ngunjiri JM, Mohni KN, Sekellick MJ, Schultz-Cherry S, Webster RG, Marcus PI. Lethal H5N1 influenza viruses are not resistant to interferon action in human, simian, porcine, or chicken cells. Nature Med. 2012;18:1456–1457. 10.1038/nm.2879 23042343

[28] Bracci L, Canini I, Venditti M, Spada M, Puzelli S, Donatelli I, et al Type I IFN as a vaccine adjuvant for both systemic and mucosal vaccination against influenza virus. Vaccine. 2006 24(Suppl. 2):56–57 16823927 10.1016/j.vaccine.2005.01.121

[29] Bracci L, Canini I, Puzelli S, Sestili P, Venditti M, Spada M, et al Type I interferon is a powerful mucosal adjuvant for a selective intranasal vaccination against influenza virus in mice and affects antigen capture at mucosal level. Vaccine 2005;23:2994–3004. 15811645 10.1016/j.vaccine.2004.12.006

[30] Le Bon A, Tough DF. Links between innate and adaptive immunity via type I interferon. Curr Opin Immunol. 2002;14: 432–436. 12088676 10.1016/s0952-7915(02)00354-0

[31] Le Bon A, Schiavoni G, D’Agostino G, Gresser I, Belardelli F, Tough DF. Type I interferons potently enhance humoral immunity and can promote isotype switching by stimulating dendritic cells in vivo. Immunity. 2001;14: 461 11336691 10.1016/s1074-7613(01)00126-1

[32] Le Bon A, Etchart N, Rossmann C, Ashton M, Hou S, Gewert D, et al Cross-priming of CD8+ T cells stimulated by virus-induced type I interferon. Nat Immunol. 2003;4:1009–1015. 14502286 10.1038/ni978

[33] Le Bon A, Durand V, Kamphuis E, Thompson C, Bulfone-Paus S, Rossmann C. Direct stimulation of T cells by type I IFN enhances the CD8+ T cell response during cross-priming. J Immunol. 2006;176: 4682–4689. 16585561 10.4049/jimmunol.176.8.4682

[34] Mueller SN, Langley WA, Carnero E, García-Sastre A, Ahmed R. Immunization with live-attenuated influenza viruses expressing altered NS1 proteins results in potent and protective memory CD8+ T cells responses. J Virol. 2010;84:1847–1855. 10.1128/JVI.01317-09 19939929 PMC2812357

[35] Nagao Y, Yamashiro K, Hara N, Horisawa Y, Kato K, Uemura A. Oral-mucosal administration of IFN-α potentiates immune response in mice. J. Interferon Cytokine Res. 1998;18:661–666. 9781804 10.1089/jir.1998.18.661

[36] Proietti E, Bracci L, Puzelli S, Di Pucchio T, Sestili P, De Vincenzi E, et al Type I IFN as a natural adjuvant for a protective immune response: lessons from the influenza vaccine model. J Immunol. 2002;169: 375–383. 12077267 10.4049/jimmunol.169.1.375

[37] Staats HF, Bradney CP, Gwinn WM, Jackson SS, Sempowski GD, Liao HX, et al Cytokine requirements for induction of systemic and mucosal CTL after nasal immunization. J. Immunol. 2001;167:5386–5394. 11673557 10.4049/jimmunol.167.9.5386

[38] Wang L, Suarez DL, Pantin-Jackwood M, Mibayashi M, García-Sastre A, Saif YM, et al Characterization of influenza virus variants with different sizes of the non-structural (NS) genes and their potential as a live influenza vaccine in poultry. Vaccine. 2008;26:3580–3586. 10.1016/j.vaccine.2008.05.001 18539366 PMC2785844

[39] Ali A, Daniels JB, Zhang Y, Rodriguez-Palacios A, Hayes-Ozello K, Mathes L, et al Pandemic and seasonal human influenza virus infections in domestic cats: prevalence, association with respiratory disease, and seasonality patterns. J Clin Microbiol. 2011;49:4101–4105. 10.1128/JCM.05415-11 21956989 PMC3233002

[40] Lee CW. Reverse genetics of the avian influenza virus. Methods Mol Biol. 2014;1161:37–50 10.1007/978-1-4939-0758-8_4 24899418

[41] Tang Y, Lee CW, Zhang Y, Senne DA, Dearth R, Byrum B, et al Isolation and characterization of H3N2 influenza A virus from turkeys. Avian Dis. 2005;49(2):207–13. 16094824 10.1637/7288-101304R

[42] Marcus PI, Ngunjiri JM, Sekellick MJ. Dynamics of Biologically Active Sub-populations of Influenza Virus: Plaque-Forming, Noninfectious Cell-Killing, and Defective-Interfering Particles. J Virol. 2009;83:8122–8130. 10.1128/JVI.02680-08 19494019 PMC2715774

[43] de Veer MJ, Holko M, Frevel M, Walker E, Der S, Paranjape JM, et al Functional classification of interferon-stimulated genes identified using microarrays. J Leukoc Biol. 2001;69: 912–920 11404376

[44] García-Sastre A, Egorov A, Matassov D, Brandt S, Levy DE, Durbin JE, et al Influenza A virus lacking the NS1 gene replicates in interferon-deficient systems. Virology 1998;252:324–330. 9878611 10.1006/viro.1998.9508

[45] Palese P, García-Sastre A. Influenza vaccines: present and future. J Clin Invest. 2002;110(1):9–13. 12093881 10.1172/JCI15999 PMC151037

[46] Ayllon J, Russell RJ, Garcia-Sastre A, Hale BG. Contribution of NS1 effector domain dimerization to influenza A virus replication and virulence. J. Virol. 2012;86:13095–13098. 10.1128/JVI.02237-12 22993153 PMC3497675

[47] Bornholdt ZA, Prasad BV. X-ray structure of NS1 from a highly pathogenic H5N1 influenza virus. Nature. 2008;456:985–988. 10.1038/nature07444 18987632 PMC2798118

[48] Nemeroff ME, Qian XY, Krug RM. The influenza virus NS1 protein forms multimers in vitro and in vivo. Virology. 1995;212:422–428. 7571411 10.1006/viro.1995.1499

[49] Salvatore M, Basler CF, Parisien JP, Horvath CM, Bourmakina S, Zheng H, et al Effects of influenza A virus NS1 protein on protein expression: the NS1 protein enhances translation and is not required for shutoff of host protein synthesis. J Virol. 2002;76:1206–1212. 11773396 10.1128/JVI.76.3.1206-1212.2002 PMC135795

