# Supplementary material for: In Vivo Assessment of NS1-Truncated Influenza Virus with a Novel SLSYSINWRH Motif as a Self-Adjuvanting Live Attenuated Vaccine
Source: PLoS One. 2015 Mar 19;10(3):e0118934. doi: 10.1371/journal.pone.0118934 (PMC4366013; doi:10.1371/journal.pone.0118934)
Supplement: S1 Fig — Areas of purple-red consolidation indicative of pneumonia are shown with arrows in the unvaccinated pig lungs. (DOCX) [file pone.0118934.s001.docx]

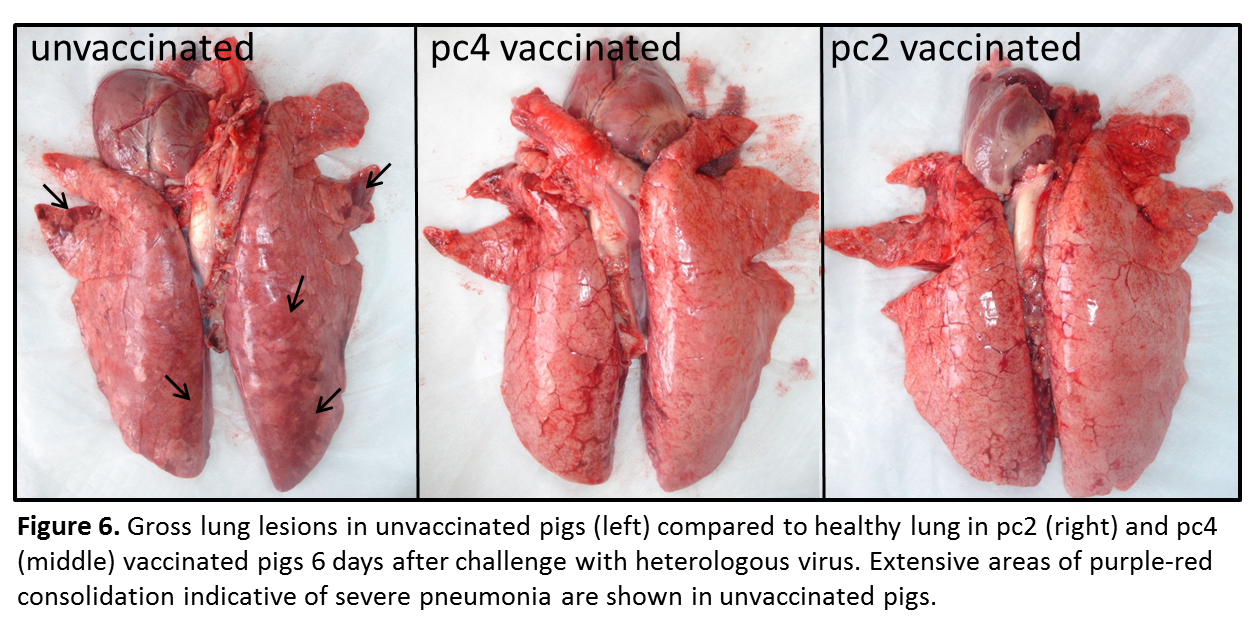


**Fig. S1.** Gross lung lesions in unvaccinated (left) compared to healthy lungs in pc2 (right) and pc4 (middle) vaccinated pigs at 6 days post challenge with heterologous virus. Areas of purple-red consolidation indicative of pneumonia are shown with arrows in unvaccinated pig lungs.
