# Supplementary material for: In Vivo Assessment of NS1-Truncated Influenza Virus with a Novel SLSYSINWRH Motif as a Self-Adjuvanting Live Attenuated Vaccine
Source: PLoS One. 2015 Mar 19;10(3):e0118934. doi: 10.1371/journal.pone.0118934 (PMC4366013; doi:10.1371/journal.pone.0118934)
Supplement: S2 Fig — Error bars represent the standard deviation from the mean (n = 4). (DOCX) [file pone.0118934.s002.docx]

**Fig S2.** Serum titers of IFN-α at 3 day post-vaccination. Error bars represent the standard deviation from the mean (*n*=4).
